# Inflammatory Pseudotumor in the Parapharyngeal Space: Is It Possible to Diagnose by Exclusion?

**DOI:** 10.7759/cureus.18907

**Published:** 2021-10-19

**Authors:** Savvas Kourtidis, Panagiotis Saravakos, Christoph Fiehn, Serena Preyer

**Affiliations:** 1 Otorhinolaryngology and Head and Neck Surgery, Charité Universitätsmedizin, Berlin, DEU; 2 Otorhinolaryngology and Head and Neck Surgery, ViDia Kliniken, Karlsruhe, DEU; 3 Rheumatology and Clinical Immunology, Medical Centre Baden-Baden, Baden-Baden, DEU

**Keywords:** follow-up, corticosteroid therapy, imaging, parapharyngeal space, skull base, head and neck, inflammatory myofibroblastic tumor, inflammatory pseudotumor

## Abstract

Inflammatory pseudotumor (IP) is a rare pathologic condition that easily can be confounded with malignancy. The clinical presentation depends on the site of occurrence and the radiological or laboratory findings are not specific. Diagnosis can be established only with histology. We report a case of a 64-year-old woman with IP in an uncommon localization, the parapharyngeal space extending to skull base. Although the diagnosis was not certain after histopathological examination, broad diagnostic workup helped to exclude malignancy or bacterial infection and led to diagnosis of an IP by exclusion. We observed a good clinical and radiological regression of symptoms after administration of oral immunosuppressants, confirming the immunological mechanism of the disease.

## Introduction

Inflammatory pseudotumor (IP) counts as a rare, benign entity and constitutes a diagnostic challenge in clinical practice. The various terms given for this condition and the different sites of occurrence are reflecting the disputable etiology and its heterogenous histopathogenesis [1,2]. Infectious, post-traumatic, or post-surgical inflammatory causes are postulated for its pathogenesis [1]. An autoimmune or paraneoplastic pathophysiologic mechanism is not yet excluded [1]. Histologically, it usually appears as a granulomatous process and comprises of (myo-)fibroblastic spindle cells, mature lymphocytes, and extracellular collagenous stroma [2]. Some authors are linking the IP with IgG4-related sclerosing disease, due to pervasive IgG4-positive lymphocyte infiltration [3]. The clinical behavior and history progression vary significantly and despite its benign histological constellation, it can imitate malignancy because of tissue infiltration and local destruction. The symptoms are depending on the site of origin; general symptoms such as fever are alternating [1].

A rare case of IP deriving from the parapharyngeal space and affecting the adjacent skull base is presented with its corresponding clinical, serological, audiological, and radiological findings. The clinical presentation as Eustachian tube dysfunction and the unusual histopathological constellation is highlighted. Two years follow-up after effective treatment with immunosuppressants is also presented.

## Case presentation

**Figure 1:**
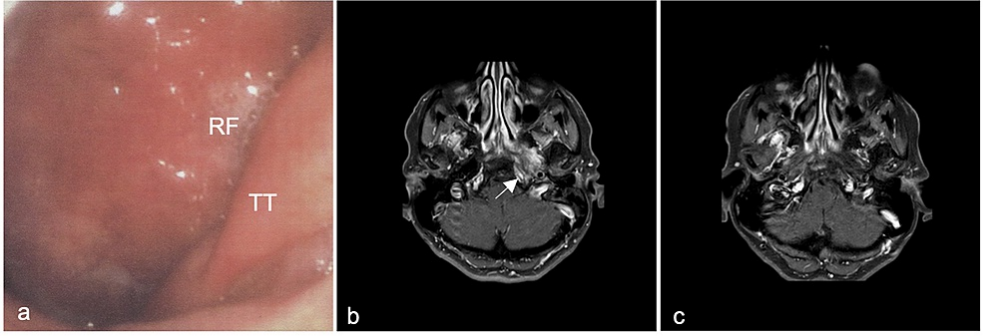
Endoscopic examination of nasopharynx, T1-weighted MRI imaging, and radiological findings after treatment. (a) Diffuse, bulging erythema of the left nasopharynx as initial finding upon endoscopic examination of nasopharynx. (b) Contrast-enhanced, T1-weighted MRI imaging, axial projection of the mass (white arrow). The mass was extending to the foramen lacerum and ovale and was surrounding the carotid canal and the Eustachian tube. The clivus was also partially infiltrated on the left side. Fluid retention in the left mastoid cavity and cervical/retropharyngeal lymphadenopathy was noted. (c) Almost complete resolution of radiological findings after treatment. TT: torus tubarius; RF: Rosenmüller’s fossa

**Figure 2:**
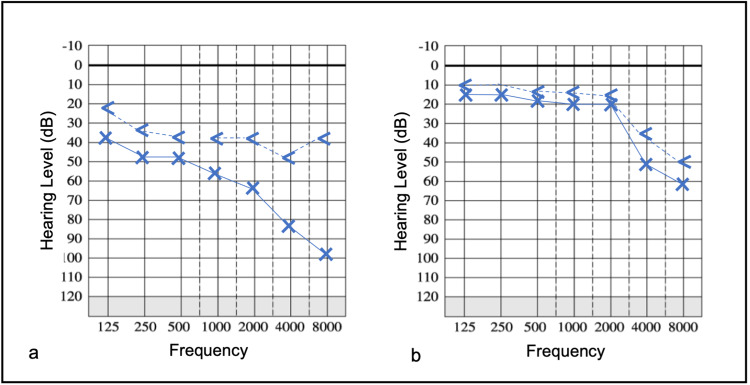
Pure tone audiometry of the left side. (a) Combined hearing loss at initial presentation and (b) improvement of hearing ability after two years of consistent therapy.

We report a case of a 64-year-old female patient who presented in our outpatient clinic with gradually evolving, left aural fullness and hearing impairment accompanied by ipsilateral tinnitus over four months. Additionally, she complained about pressure over the left temporal region. She had no significant medical and family history. Upon examination, we noted a left middle ear effusion and a diffuse, bulging, mucosal erythema of the nasopharynx on the left side (Figure 1, panel a). The pharyngeal orifice of the Eustachian tube appeared normal. Cranial nerve examination revealed no abnormalities, palpation of the neck showed no mass or tenderness. Laboratory tests showed a slightly elevated erythrocyte sedimentation rate (Table 1). Pure tone audiometry showed a combined hearing loss on the left side (Figure 2, panel a). Contrast-enhanced magnetic resonance imaging (MRI) of skull base revealed a T1- and T2-weighted hypointense mass of the left parapharyngeal space with indistinct margins and strong, homogenous gadolinium uptake (Figure 1, panel b). The corresponding computed tomography scan (CT) showed discrete erosion of the cortical bone of the middle cranial fossa.

**Table 1:** Relevant laboratory findings during diagnostic workup.

Test		Reference	Unit	
Leukocytes	6.5	3.3-10.7	/nl	
C-reactive protein	<1	0-5	mg/l	
Erythrocyte sedimentation rate (ESR)	50	25	mm/h	
Soluble IL-2 receptor	674	623	U/l	
Angiotensin-converting enzyme (ACE)	49.8	20-70	U/l	
Tuberculosis enzyme-linked immunospot (ELISPOT)	Negative			
Rheumatoid factor IgA	<20	<20	IU/ml	
Anticitrullinated protein IgG (antiCCP)	Negative			
Antineutrophil cytoplasmic antibodies (ANCA): cytoplasmic (cANCA), perinuclear (pANCA),	Negative			
Antineutrophil cytoplasmic antigens: proteinase 3 antigen (PR3), myeloperoxidase (MPO)	Negative			
Antinuclear antibodies (ANA)	Negative			
Extractable nuclear antigen (ENA)	Negative			
IgG	1484	700-1600	mg/dl	
IgG1	994	405-1011	mg/dl	
IgG2	364	169-786	mg/dl	
IgG3	41.3	11-85	mg/dl	
IgG4	84.9	3-201	mg/dl	
Epstein-Barr virus-antivirus capsid antigen-IgG (EBV-antiVCA-IgG)	750	20	U/ml	
Epstein-Barr virus-antivirus capsid antigen-IgM (EBV-antiVCA-IgM)	10	20	U/ml	
Epstein-Barr virus-antiearly antigen-IgG (EBV-antiEA-IgG)	97	40	U/ml	

Since a malignant tumor was suspected, a transoral nasopharyngeal biopsy was performed primarily. The mucosal probe was taken by a bended double-cup ended forceps. The histological examination revealed typical benign features of respiratory epithelium with submucosal lymphoid follicles. This result was interpreted as “sampling error” and therefore a second, transnasal, endoscopically guided, incisional re-biopsy was conducted. The histological evaluation delivered the same result as above, despite multiple and adequate probes from deep layers. The futile biopsies led to an extended diagnostic workup to rule out the differential diagnoses, such as tuberculosis, sarcoidosis, and antineutrophil cytoplasmic antibodies (ANCA)-associated diseases such as M. Wegener were excluded with appropriate serological tests and pulmonological consultation and imaging (Table 1). The patient consented to a trial antibiotic course with per os clindamycin, 600 mg three times per day, under the assumption of an infectious cause and a referral to a university otolaryngologic clinic for obtaining a second opinion. There, a third transnasal-endoscopically, CT-guided core biopsy, and comprehensive histopathological examination did not provide any additional information about the entity. Antibiotics did not resolve the symptoms nor the radiological signs over a period of two months, thus, a review of the diagnosis was necessary. The consulted rheumatologist proposed a probatory corticosteroid and immunosuppressive therapy as the differential diagnoses were narrowed down to immunological diseases, the diagnosis of IP became plausible. Fortunately, our patient responded well, symptoms disappeared during the first weeks of treatment, and radiological signs resolved gradually in almost four months. The therapeutic scheme included initially per os prednisolone 20 mg once per day and subcutaneous methotrexate 10 mg once weekly (body weight: 48 kg), the corticosteroid dose was sequentially reduced down to 2 mg per day. The patient was followed up for nearly two years and is currently symptom-free. The radiological findings dissolved almost completely except for discrete residual spots of gadolinium enhancement in the parapharyngeal space (Figure 1, panel c). The combined hearing impairment was also regressive (Figure 2, panel b). Several attempts to completely stop prednisone treatment resulted in reappearance of initial symptoms such as aural fullness and pressure. Therefore, we decided to remain on stable doses of methotrexate 10 mg per week plus prednisolone 2 mg per day further on.

## Discussion

Although IP can manifest in various sites and is depicted with different terminology, the lung and the orbit are most frequently affected [1] (Table 2). With only nine cases of IP of the parapharyngeal space previously published, this entity has to be characterized as exceptionally seldom [4-8]. Nevertheless, it should be considered in the differential diagnosis for lesions in this region, particularly in uncertain cases [9].

**Table 2:** Various names and sites for inflammatory pseudotumor (IP) after Narla et al. [1].

Names of IP	Sites for IP
Plasma cell granuloma	Trachea-lung-diaphragm
Inflammatory myofibroblastic tumor	Orbit
Inflammatory myofibrohistiocytic proliferation	Nasal cavity-paranasal sinuses-nasopharynx
Histiocytoma	Testis
Xanthoma	Liver-spleen-pancreas
Fibroxanthoma	Bladder-kidney-adrenal gland
Xanthogranuloma	Mesentery-retroperitoneum
Fibrous xanthoma	Heart
Xanthomatous pseudotumor	Thyroid
Plasma cell-histiocytoma complex	Central nervous system-meninges
Plasmocytoma	Gastrointestinal
Solitary mast cell granuloma	Tonsil
Inflammatory fibrosarcoma	

Our patient presented with symptoms of obstructive Eustachian tube dysfunction. Initially, we suspected a malignant mass of the parapharyngeal space giving rise to middle ear effusion and ipsilateral dull pain of the temporal region. Furthermore, the lesion led to a combined hearing loss, affecting the sensorineural function of the inner ear. The pathophysiologic mechanism of the sensorineural hearing loss is unclear, toxic labyrinthitis due to persistent middle ear effusion could be an explanation. In contrast to the case report of Maruya et al., the infiltration of the foramen ovale and lacerum did not cause any cranial nerve palsies [7]. Fever was not part of the clinical presentation as reported in other cases [1,8].

The radiologic characteristics of IP are various and not specific, they can mimic the appearance of inflammatory or malignant processes [10]. In the present study, repeated MRI imaging combined with close clinical monitoring and extensive serological workup helped us to delineate the diagnosis by exclusion. Several transnasal biopsies, even assisted with CT-navigation, were unsuccessful in yielding pathognomonic characteristics, such as positive IgG4 immunochemistry. The serological tests for immunological diseases (for example, M. Wegener, sarcoidosis) and association with IgG4 syndromes (IgG4 serum levels) were negative. It remains unknown, whether a more invasive surgical approach, such as a transcervical transparotideal approach, would have allowed a more representative biopsy [4,7].

The establishment of the correct diagnosis is crucial to administer proper therapy and avoid unnecessary morbidity through surgical procedures. We primarily started a trial, long-term antibiotic course; after two months of close clinical and radiological monitoring during antibiotic regime, we noted neither a progression nor a regression of the lesion. Since the pathology did not respond to the antibiotic treatment, we ruled out an infectious osteomyelitis. This fact was suggesting an autoimmune inflammatory process. The probatory immunosuppressive therapy with corticosteroids and methotrexate showed a tremendous response and led to almost complete resolution of symptoms and radiological signs of the tumor in four months. Other authors reported primarily surgical or combined therapeutic options with good level of control [5,8].

## Conclusions

Despite the fact that the diagnosis of IP should rely on clinical-histopathological criteria, a high level of alertness of presence of this entity in uncommon sites could turn out advantageous for the clinician. It is crucial to rule out malignant and infectious disease at first glance. Even without a pathognomonic histopathological and serological constellation, IP should be considered as a differential diagnosis and be treated with antiinflammatory and immunosuppressive therapy.
